# Bleeding “sarcomatosis” as a rare presentation of vascular graft-related angiosarcoma: case report and review of the literature

**DOI:** 10.1186/s12893-020-00966-7

**Published:** 2020-11-20

**Authors:** Stefano Presacco, Amedea L. Agnes, Sabina Magalini, Arnaldo Carbone, Maurizio Martini, Annamaria Agnes

**Affiliations:** 1 Università Cattolica del Sacro Cuore, Largo Francesco Vito n. 1, 00168 Rome, Italy; 2 Dipartimento Scienze Mediche e Chirurgiche, Fondazione Policlinico Universitario Agostino Gemelli IRCCS, Largo Agostino Gemelli 8, 00168 Rome, Italy; 3 Dipartimento Scienze della Salute della Donna, del Bambino e di Sanità Pubblica, Fondazione Policlinico Universitario Agostino Gemelli IRCCS, Largo Agostino Gemelli 8, 00168 Rome, Italy

**Keywords:** Angiosarcoma, Sarcomatosis, Vascular-graft, Case report

## Abstract

**Background:**

Angiosarcoma is a rare malignant tumor, originating from vascular endothelial cells, accounting for approximatively 1–2% of soft tissue sarcomas. It is characterized by a rapid proliferation and high metastatic potential. Some cases of angiosarcoma are described in association with vascular prosthesis, orthopedic devices and foreign bodies. Hereby, we report a case of a patient treated with the endovascular placement of a PTFE aorto bis-iliac prosthesis for aortic aneurysm, who developed a graft-related angiosarcoma with bone and peritoneal localizations. The peritoneal “sarcomatosis” led to an acute presentation with hemoperitoneum and anemia. We perform a thorough review of the literature summarizing the description of similar cases, their epidemiology and the possibilities for treatment.

**Case presentation:**

An 84-year-old male with a history of abdominal aortic aneurysm endovascular repair presented to our emergency department complaining with low back pain radiating to the left limb. He underwent a type II endoleak embolization of the aneurysmal sac nine days before. During hospitalization he underwent a spine MRI which documented a vertebral alteration of non-univocal interpretation. Vertebral biopsy was performed revealing groups of cells of uncertain nature. He lately underwent percutaneous L2–L4 arthrodesis. Forty-two days after admission, he developed acute anemia. Emergency laparotomy revealed a massive hemoperitoneum and actively bleeding peritoneal nodules. Abdominal packing was performed, and several nodules were sent for definitive histological examination. After surgery, he developed progressive and severe hypovolemic shock and expired on postoperative day 5.

**Conclusions:**

Angiosarcoma associated with foreign bodies, especially vascular prosthesis, is a very rare entity. In patients who have a history of prosthetic vascular graft placement that present with lumbar pain, osteolytic changes at radiologic imaging or the development of ascites, angiosarcoma should be considered in the differential diagnosis. Despite the poor prognosis, a prompt diagnosis might give access to an adequate treatment planning, with the aim for disease control and increased survival.

## Background

Angiosarcoma (AS) is a rare malignant tumor, accounting for 1–2% of soft tissue sarcomas [1, 2]. It arises from the endothelial cells of the blood or lymphatic vessels, and mainly occurs in the skin and superficial soft tissues. Less frequently, it occurs in deep soft tissues, like liver, heart, bone and spleen [1, 2]. Its occurrence in the gastrointestinal tract is extremely rare, and the development of hemoperitoneum is even rarer [1–4].

Due to the difficulties associated with the diagnosis and its aggressive nature, AS is often diagnosed at an advanced or metastatic stage [1, 2]. For the differential diagnosis, histological findings including immunohistochemistry are most important [1, 3]. Some cases of AS are described in association with vascular prosthesis and orthopedic devices [5–7].

In this report, we describe the case of an 84-year old male with a history of abdominal aortic aneurysm (AAA) treated by an endovascular procedure, who developed an AS with intra-peritoneal dissemination that unusually presented with acute anemia and hemoperitoneum.

## Case presentation

**Fig. 1:**
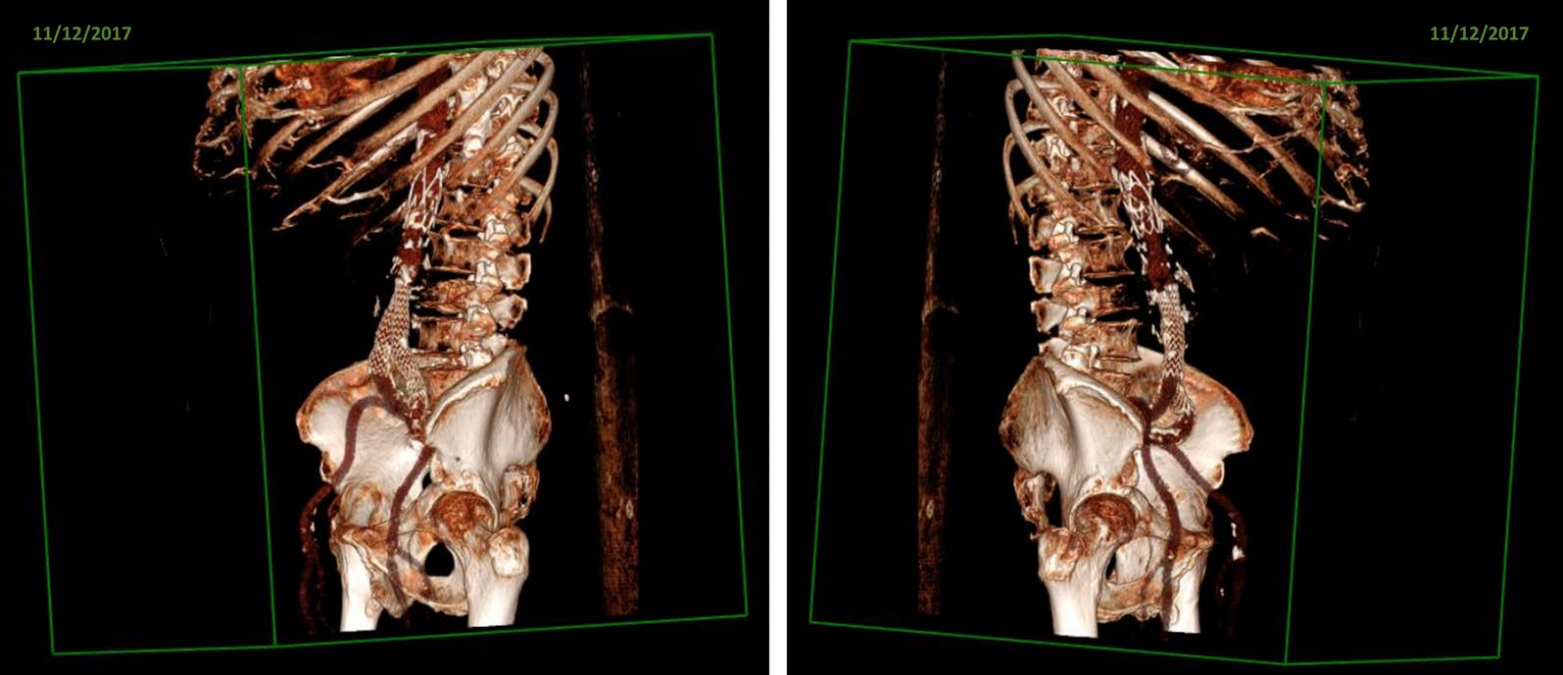
Volume rendering CT reconstruction showing the endoprosthesis

An 84 years-old male in good general conditions was admitted to the Emergency Department in November 2017 complaining of lumbar pain radiated to the left lower limb. Nine days before, he underwent an embolization procedure for a type II endoleak of the aneurysmal sac in another hospital. In 2012, he had undergone the endovascular placement of anOvation Abdominal Stent Graft System (TriVascular Inc., Santa Rosa, CA) polytetrafluoroethylene (PTFE) aorto bis-iliac endoprosthesis for an abdominal aortic aneurysm (AAA) (Fig. 1). He had a history of hypertension, non-significant carotidal vasculopathy, dyslipidemia, colonic diverticular disease and benign prostatic hyperplasia. He had previous inguinal hernia surgery and appendectomy. He was a former smoker. Since 2 months, he had developed a constant lumbar pain. He had undergone a magnetic resonance imaging (MRI) that documented a cystic/osteolytic appearance of the vertebral body of L3 with possible partial collapse. The pain worsened abruptly after discharge from the hospital where he had the recent endovascular procedure.

**Fig. 2:**
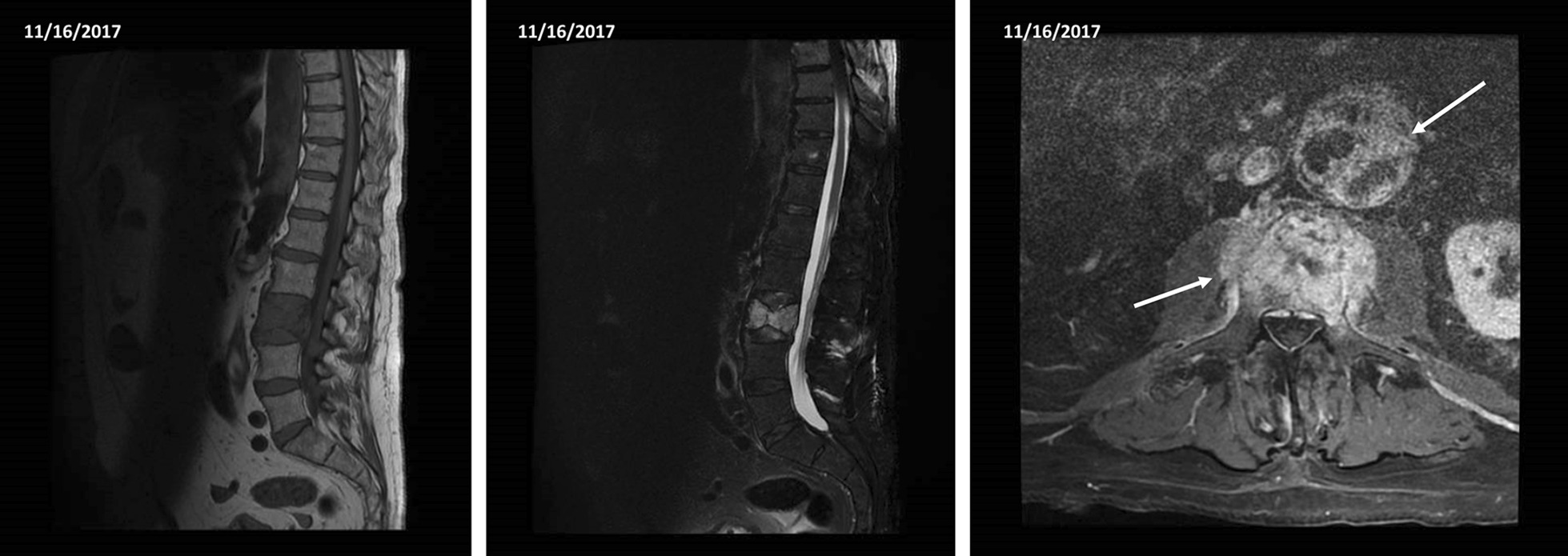
MRI imaging showing the vertebral alteration L3 and the inhomogeneous enhancement of the aortic sac

**Fig. 3:**
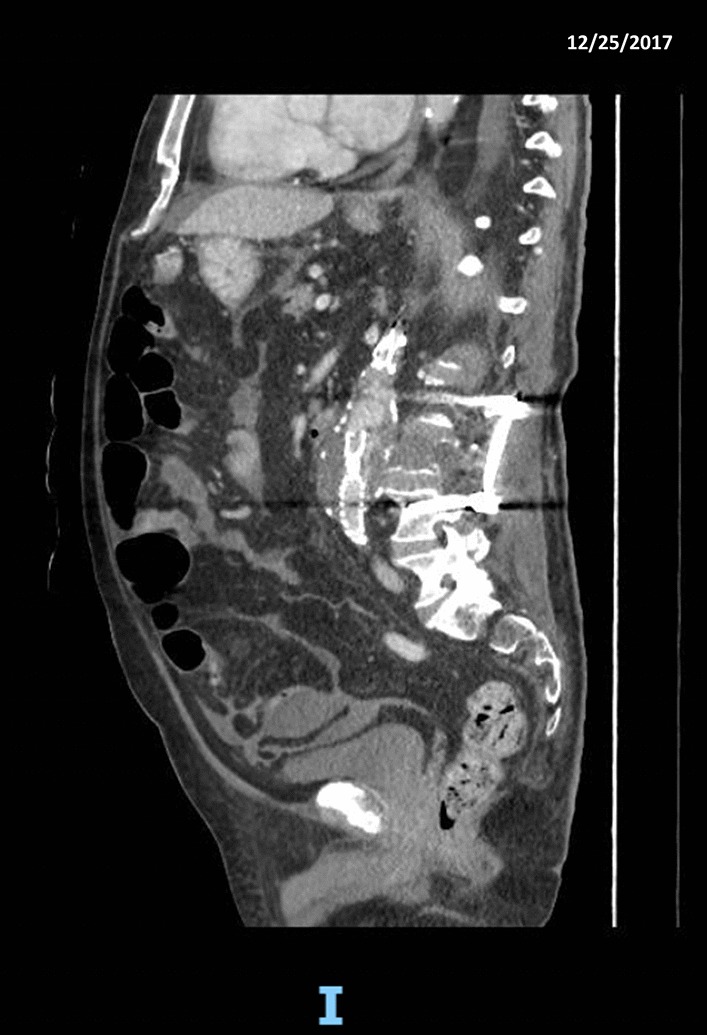
CT imaging showing the contiguity between the aortic sac and the affected vertebra (after the L2–L4 arthrodesis)

On admission in our hospital, his blood pressure and heart rate were within the normal range. The patient had intense lumbar pain unresponsive to analgesics. Laboratory workup was non-significant. Hemoglobin was 12.6 g/dL. An abdominal computed tomography(CT) scan was performed. It documented an L3 vertebral collapse of uncertain nature (neoplasms or osteonecrosis). No signs of endoleak were detected. The patient was admitted to the Geriatrics department for further management. In order to exclude a secondary bone lesion a skull/chest CT scan was ordered. The CT scan did not report any primary neoplasms. Tumor markers (CYFRA 21-1, CEA, CA 19-9, CA 15-3, alfa-FP, PSA, neuron-specific enolase, CA 125) were negative. A new MRI documented a vertebral alteration of non-univocal interpretation, increased in dimension when compared with the previous MRI. Some areas of the aneurysmal sac showed an inhomogeneous enhancement (Fig. 2). The main clinical suspicions were a neoplasm, osteonecrosis or septic spondylitis. Seven days after admission, he developed a fever (38 °C). He had no leukocytosis. CRP was 160 mg/dL, procalcitonin was negative (0.24 ng/mL). Blood cultures were positive for *Serratia marcescens* and urine culture tested positive for *Enterobacter cloacae* and *Enterococcus faecalis*. Quantiferon test for tuberculosis was negative. The patient was treated with intravenous antibiotic therapy (Ceftriaxone and Levofloxacin) and the fever subsided. Three days later, he was transferred to the Vertebral Surgery department and underwent a transpedicular percutaneous L3 biopsy. Pathological examination documented the presence of fibrino-hematic material and rare small groups of cells with irregular profile and nucleus of uncertain nature. The microbiological examination was negative. Twenty-two days after admission, a percutaneous L2–L4 arthrodesis was performed (Fig. 3).

**Fig. 4:**
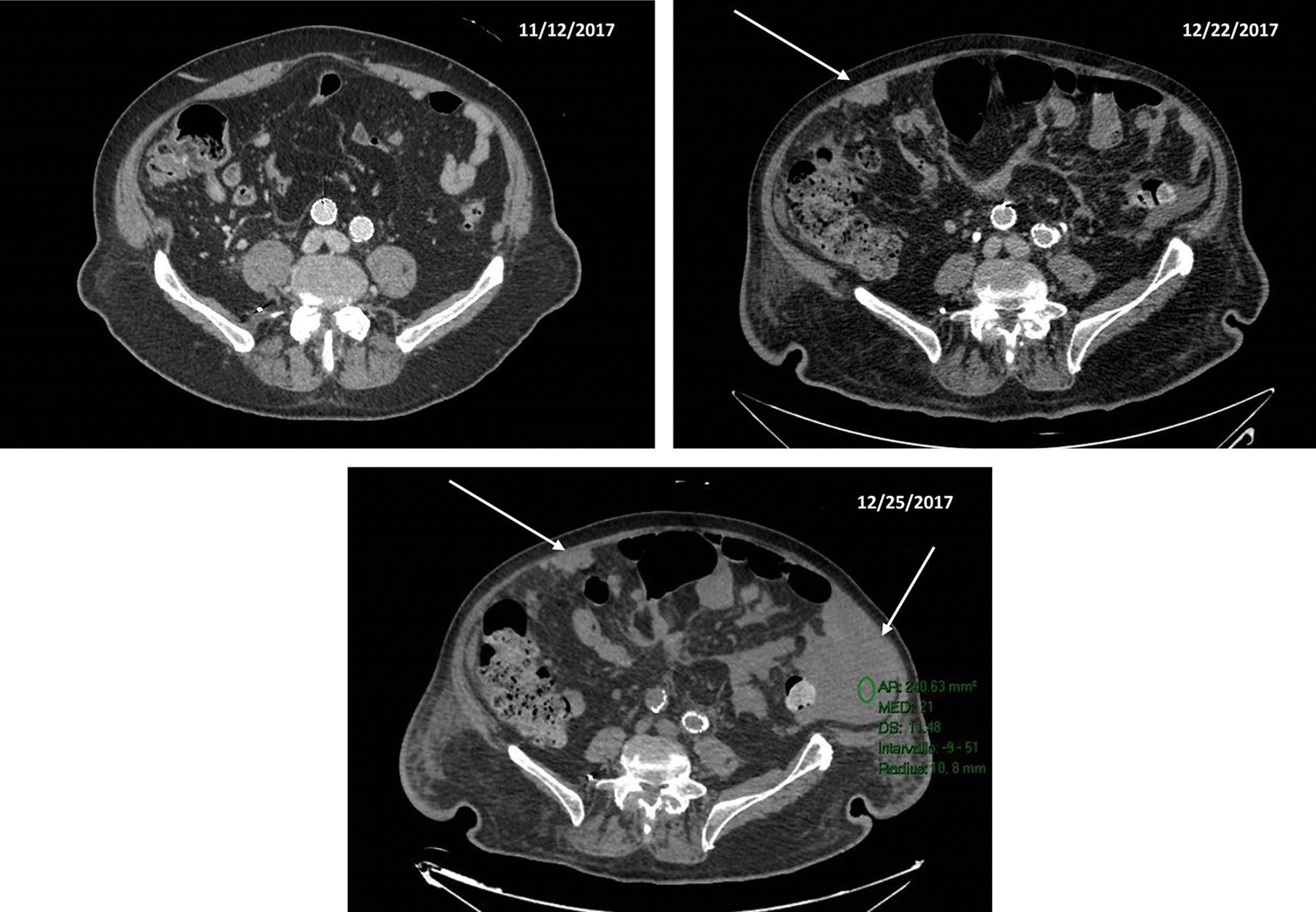
CT imaging showing the rapid evolution of the peritoneal localizations and ascites

During hospitalization, the patient developed progressively severe anemia. Hemoglobin was 10.8 g/dL on the 19th day after admission, 9.6 g/dL on the 22nd day after admission, 7.6 mg/dL on the 25th day after admission. The patient was hemodynamically stable. He complained of slight abdominal pain, in the absence of hematemesis or melena (stools were normocromic). The patient was transfused with 1 unit of red blood cells (RBCs). At post-tranfusional controls, hemoglobin remained stable (Hb 9.1 g/dL). Twenty-eight days after admission, the patient was transferred to the rehabilitation service of the hospital. He complained of some lumbar pain manageable with analgesics. Thirty-nine days after admission, a follow-up CT scan showed a slight enhancement close to the iliac extremities of the endoprosthesis, suspect for endoleak. The peritoneal fluid detected at the previous CT scan was notably increased (Fig. 4). The alteration of the vertebral body of L3 was stable. Forty-two days after admission, the patient developed acute anemia (hemoglobin 7.7 mg/dL), hypotension (70/40 mmHg) and tachycardia (170 bpm). Three units of RBCs were transfused and CT-angiography was performed. The exam documented a large volume of ascitic effusion arranged in the perihepatic and perisplenic space, between the intestinal loops and in the pelvis. The ascites had localized increased density due to the presence of corpuscular material. Thickening of the mesenteric sheets and the presence of some formations of irregular morphology, with soft tissue density, lining the parietal peritoneum in correspondence of the anterior abdominal wall were noticed. After transfusions, the patient was hemodynamically stable. No fever was reported. At clinical examination, the abdomen was distended but non-tender. Bowel function and stools were normal. Laboratory workup was significant for INR 1.23 (0.80–1.20), aPTT 45.2 s (20.0–38.0), antithrombin III activity 67% (70–140), creatinine 1.78 mg/dL (0.67–1.17 mg/dL), hemoglobin 7.5 g/dL, white blood cell count 12.910/mm^3^. On the next day, a 6 Fr pigtail catheter was placed by ultrasound (US) guidance in the right hypochondrium. Twenty mL of bloody ascites were drawn and sent in for microbiological and biochemical examination (negative) and for cytological examination (negative for lack of material). In a few hours, the output of the drain increased to 1500 mL of blood. The patient became hemodynamically unstable. Surgical evaluation posed an indication for emergency laparotomy. At the opening of the abdomen, 3 L of blood were drained. Numerous blackish nodules were found on the intestinal loops and lining the omentum. Some of them were actively bleeding. The main source of bleeding was apparently venous and came from the abdominal wall in the suprapubic region, where a nodular omentum was tenaciously attached to the abdominal wall. Hemostasis was carried out with diathermocoagulation and a fibrillar hemostatic agent. Three nodules were sent for definitive histological examination. Abdominal packing was performed. After surgery, the patient was transferred to the Intensive Postoperative Care Unit. He was transfused with RBCs, fresh frozen plasma, albumin, crystalloid fluids (3000 mL). His general conditions were critical, with hypotension, anemia and leukocytosis. He needed inotropic support. On the 4th postoperative day, a new output of blood was observed from the surgical drain. Due to the poor general conditions, no surgical maneuver was planned. He subsequently developed severe hypovolemic shock with anuria and hypotension and expired on the 5th postoperative day (49 days after admission).

**Fig. 5:**
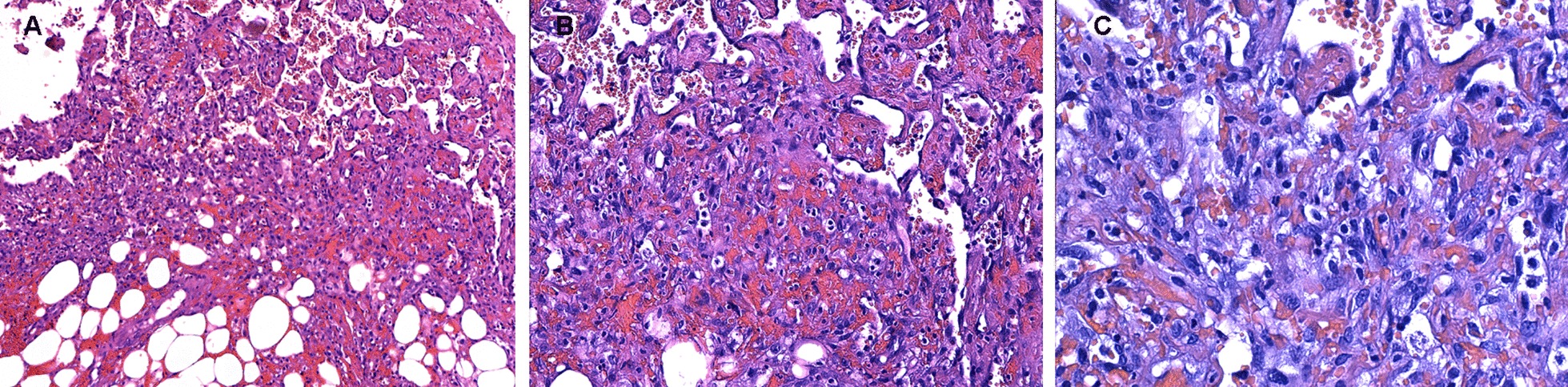
Histologic examination of the peritoneal samples. Hematoxylin and eosin (H&E) staining, original magnification Panel **a**: AX100, Panel **b**: X200, Panel **c**: X 400

**Fig. 6:**
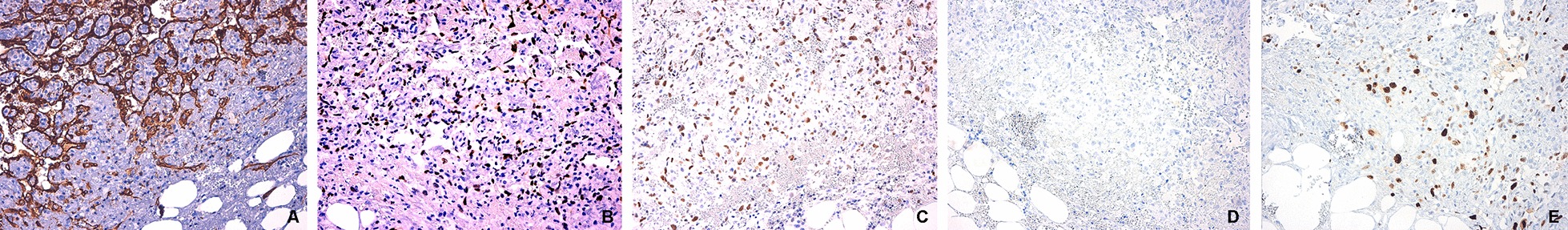
Histologic examination of the peritoneal samples. Immunoistochemistry, original magnification X200 Panel **a**: CD31, Panel **b**: ERG, Panel **c**: FLI1, Panel **d**: HHV8; Panel **e**: Ki67

Definitive pathological examination documented hematogenous material in the form of collections infiltrating the adipose omental tissue, which showed to be densely populated by thin-walled entangled vessels, with a high density of the endothelial cells. Endothelial cells had hyperchromic, polymorphic nuclei and there was focal atypia. No mitosis was documented. In some parts of the adipose tissue, the epithelial elements were organized in a micropapillary structure. Immunohistochemistry for CD31, ERG, FLI-1 confirmed the endothelial nature of the elements described (and resulted unremarkable for HHV8 antigen). The endothelial elements were actively proliferating according to Ki67 immunostaining. The calretinin staining showed small superficial areas covered by peritoneum. The examination was consistent with epithelioid angiosarcoma (EA) (Fig. 5, 6).

## Review of the literature

AS represents a subset of malignant tumors arising from vascular and lymphatic endothelial cells and accounts for 1–2% of soft tissue sarcomas. AS is often diagnosed at an advanced stage and has a poor prognosis, significantly lower respect other subtypes of soft tissues sarcomas [1–4]. The diffuse peritoneal spreading of primitive intra-abdominal sarcomas is defined as peritoneal sarcomatosis and represents a notable part of the history of these tumors, occurring both at the time of the first diagnosis and at advanced stages of disease [8]. Differential diagnosis between peritoneal sarcomatosis and the more common peritoneal carcinomatosis is difficult, but some clinical and radiological features may help to distinguish them. The presence of hypervascularity, hemoperitoneum with absence or small amount of ascites, bulky spherical heterogeneous deforming masses are suggestive of peritoneal sarcomatosis [9].

AS is more often primary, but it has also been frequently described as secondary soft tissue sarcoma, with relation to chronic lymphedema, radiation for breast cancer or other malignancies, familial syndromes and exposure to chemicals [1–3]. In some reports, it has been related to foreign bodies presence or implants, in particular vascular grafts and orthopedic prosthesis and devices [5–7]. Other sporadic cases were related to retained surgical sponge, bone wax, subcutaneous cardiac defibrillator and metallic bullets [10–13].

While a direct connection between foreign material and development of sarcomas (including angiosarcomas) is not clearly proven in humans, it is well established in animal models. In early studies on mice, Oppenheimer et al. demonstrated the induction of sarcoma in the fibrous pocket developing around subcutaneous embedded plastic films [14]. Karp et al. established the tumorigenic effect of subcutaneous implants whose pore size was less than 0.2 µ, leading to the formation of a thick fibrous capsule rather than a cellular inflammatory response with less fibrosis, typical of larger-pore non tumorigenic materials [15]. Another hypothesis for the vascular graft-associated cases might be the alteration of blood flow with creation of turbulence, as reported in rare cases of arteriovenous fistulae associated AS in absence of foreign material [16].

In the literature, 17 cases of aortic graft-related AS are described (Table 1) [7, 17–31], 12 of which associated with a polyester prosthesis (Dacron) [7, 17–23, 25, 26, 28, 31], and two linked to a PTFE graft [29, 30], while three cases do not mention the graft material [24, 27]. According to this review, male gender was predominant (15 cases over 17) and the mean age of disease manifestation was 64.6 years, ranging from 50 to 84 years. The timespan between the graft procedure and the occurrence of AS ranged from 3 months to 17 years, with a mean interval time of 7.9 years. Abdominal pain, back pain, fatigue and weight loss were commonly present at various grades at the time of the diagnosis in all reports. Less common symptoms and signs were unilateral lower limb pain, fever, sciatic nerve pain, claudication, palpable mass, nausea and vomiting. Further uncommon manifestations were cerebral symptoms, night sweats, tarry stools and painful skin eruption with erythematous and purpuric papules and nodules. Two cases reported peritoneal sarcomatosis without ascites development [19, 29]. One case reported a retroperitoneal hemorrhage during the clinical course, treated with surgery [23]. Moreover, none of the cases had an onset with major intra-abdominal bleeding or acute anemia at any stage of disease.

**Table 1:** 17 reported cases of aortic graft-related angiosarcoma

Author/year	Age/gender	Type of graft	Interval to AS (years)	Presenting symptoms	Histological pattern
Fehrenbacher et al., 1981 [14]	67/Male	Dacron	12	Progressive right foot pain	Angiosarcoma
Weiss et al., 1991 [15]	56/Male	Dacron	3.5	Abdominal and low back pain, sciatica	Epithelioid angiosarcoma
Ben Izhak et al., 1999 [16]	71/Male	Dacron	8	Abdominal pain, nausea, vomiting, weight loss	Epithelioid angiosarcoma
Okada et al., 2004 [17]	50/Male	Dacron	17	Cerebral symptoms	Epithelioid angiosarcoma
Umscheid et al., 2007 [18]	50/Male	Dacron	4.6	Abdominal pain, weight loss, tarry stools, palpable mass	Epithelioid angiosarcoma
Almeida et al., 2011 [19]	60/Female	Dacron	9	Upper back pain, dyspnea, fatigue, weight loss	Angiosarcoma
Schmehl et al., 2012 [20]	84/Male	Dacron	8	Abdominal pain, fatigue, weight loss	Epithelioid angiosarcoma
Fatima et al., (case 3), 2013 [21]	57/Male	?	6	Low back pain, fever, night sweats, weight loss	Epithelioid angiosarcoma
Fenton et al., 2014 [22]	66/Male	Dacron	6	Low back pain, weight loss	Angiosarcoma
Kimura et al., 2015 [23]	78/Male	Dacron	16	Fever	Angiosarcoma
Kamran et al., (case 1), 2016 [24]	69/Male	?	8	Abdominal pain, weight loss, anorexia, palpable mass	Epithelioid angiosarcoma
Kamran et al., (case 3), 2016 [24]	72/Female	?	0.25	Low back pain	Angiosarcoma
Tiwari et al., 2016 [25]	64/Male	Dacron	7	Fever, dyspnea, weight loss, painful skin eruption	Epithelioid angiosarcoma
Milite et al., 2016 [26]	60/Male	PTFE	7	Abdominal and low back pain, fever, fatigue	Epithelioid angiosarcoma
Dietl et al., 2018 [7]	59/Male	Dacron	15	Low back pain, palpable neck mass	Epithelioid angiosarcoma
Yu et al., 2019 [27]	68/Male	PTFE	4	Abdominal and low back pain, fever	Epithelioid angiosarcoma
Derouane et al., 2020 [28]	68/Male	Dacron	3.5	Claudication	Epithelioid angiosarcoma
Current case	84/Male	PTFE	5	Low back pain radiated to the left lower limb	Epithelioid angiosarcoma

*AS* angiosarcoma

Widespread metastasis were present in many cases, either at the time of the diagnosis or occurring during follow-up (mostly within 12 months), confirming the clinical aggressiveness and metastatic potential characterizing AS [7, 25]. In particular, five patients presented with spine metastasis at the time of the diagnosis [7, 22, 23, 25, 28], and three patients developed vertebral metastasis within seven months from the first symptoms [17, 30, 31].This case presented with a symptomatic vertebral involvement that needed surgical percutaneous arthrodesis and raised an initial clinical suspicion for a primary/secondary spine tumor, osteonecrosis and septic spondylitis rather than an infiltrating AS originating from the nearby aortic wall. In fact, primary AS of the bone is uncommon, accounting for less than 1% of angiosarcomas, and its primary occurrence in the spine is exceedingly rare, being documented only in few reports. Vertebral bodies at the thoracic and lumbar level are generally involved [32, 33]. Spinal AS symptoms are largely unspecific and are related to its anatomical location, presenting with radiculopathy and neurological deficits, although the first symptom is usually a generic dull back pain [32, 33]. Bone osteolysis and destruction, ill-defined margins and erosion of the cortex with soft tissue involvement are the non-pathognomonic radiological signs spinal AS shares with most of the other spine malignancies [33].

The differential diagnosis for AS strongly depends on its location and its multifaceted clinical presentation. For instance, vascular graft related AS is often misdiagnosed as pseudoaneurysm or graft infection or osteomyelitis (as it happened in our case) until a biopsy is performed, delaying the diagnosis [5]. A biopsy is always necessary to confirm the radiologic suspect [34]. The most frequent subtype of AS associated to vascular prosthesis is EA [7]. In our analysis EA appeared to be the most common histologic type (involving 12 patients out of 17), the remaining consisting of various grades of AS. EA is an aggressive subtype of AS in which malignant cells have a prevailing epithelioid aspect. It is more common in males, has the highest incidence in the seventh decade and carries a bad prognosis, with a 50% survival rate within 3 years [35]. Differential diagnosis is very important, with immunohistochemistry playing an important role in distinguishing EA from other forms like epithelioid sarcoma, metastatic carcinoma, malignant mesothelioma and less aggressive vascular neoplasms, like epithelioid hemangioendothelioma [35].

## Diagnosis and treatment

For local staging, MRI and CT scan are the gold standard, while for complete clinical staging, a chest CT is considered necessary. EA and other angiosarcomas have also a potential for diffusion by lymphatic route [1, 36], and bone metastases have been previously described for vascular graft related AS [22]. Brain CT scan is also suggested for the staging of angiosarcomas due to their tropism for brain metastases [36].

Treatment options for angiosarcoma are summarized in Table 2. The standard of care for localized non-metastatic AS, as for other sarcomas, is represented by surgery with a broad and adequate margin of resection, in addition to radiation therapy with large doses (> 50 Gy) in the adjuvant setting to improve local control [1, 36]. The management of metastatic disease, instead, relies on chemotherapy. AS is usually anthracycline-sensitive, therefore, in most institutions, an anthracycline-based regimen is the preferred first-line treatment. The peculiarity of angiosarcomas, in comparison with other types of sarcomas, is the sensitivity to single agent taxanes, with good response rates and reports of prolonged disease control. Taxanes are alternatively used a first-line or second line one depending on the institution [34]. Tyrosine kinase inhibitors as Sorafenib also showed some benefits in selected patients and they may be added to conventional cytotoxic agents, but their use is still under study [1, 37, 38]. In fact, palliative chemotherapy is complicated with toxicities and low response rates [1]. A possible treatment option for patients with good performance status, with peritoneal metastases in absence of systemic disease, is cytoreductive surgery followed by hyperthermic intraperitoneal chemotherapy. However, this treatment is very aggressive and remains controversial due to its unclear efficacy in different subsets of sarcoma [8] (Table 2).

**Table 2:** Treatment options for AS by stage of disease [1, 34, 37, 38]

Stage	Treatment
Localized disease	Surgical resection with adequate margin (R0) and adjuvant radiotherapy with large doses (> 50 Gy)
Metastatic disease	Chemotherapy with anthracyclines or taxanes as single cytotoxic drugs Biological therapies with tyrosine kinase inhibitors showed benefits (under investigation) Cytoreductive surgery and HIPEC for peritoneal metastases in the absence of systemic disease (under investigation)

*AS* angiosarcoma, *HIPEC* hypertermic intraperitoneal chemotherapy

## Discussion

To our knowledge, this is the first case in literature of vascular graft-related AS presenting with bleeding sarcomatosis, hemoperitoneum and ascites. In this patient, the main initial symptom was lumbar pain, because of the vertebral osteolysis. It is unclear if the vertebral involvement was a primary or a secondary localization of AS. A neoplastic vertebral lesion was suspected from the MRI performed after admission, although it was not clearly confirmed by the radiological workup and the pathological examination. The inhomogeneous enhancement of the aneurysmalsac at the MRI was considered an unspecific sign of possible graft infection rather than a tumor-related feature. Then, during recovery from the urinary sepsis and the vertebral stabilization, the disease progressed rapidly to a metastatic stage with peritoneal involvement. In this case, only a high index of suspicion in the evaluation of the vertebral osteolysis, linked to the history of endovascular AAA repair could have led to the challenging diagnosis of AS. However, analyzing the diagnostic process, a vertebral biopsy should probably have been repeated and integrated with immunohistochemichal staining to provide more detailed information. Still, the rapid progression of the disease would have probably not been controllable with therapy, given the clinical aggressiveness of the disease, the age of the patient and his intercurrent clinical problems. In the late phase of the hospital stay, the onset of acute anemia, hemoperitoneum and hyperdense ascites was considered more suggestive of ruptured aortic aneurysm than of peritoneal sarcomatosis and the diagnosis was challenging as well. However, this case is educative to raise the clinical suspicion for prosthesis-related AS and increases the number of cases reported in literature.

## Conclusion

In conclusion, AS associated with foreign bodies, especially vascular prosthesis, is a very rare entity. In patients who have a history of prosthetic vascular graft placement that present the occurrence of lumbar pain, osteolytic changes at the radiologic imaging or the development of ascites, AS should be considered in the differential diagnosis. The prognosis of this disease is usually poor. However, a prompt diagnosis of AS, even at an advanced stage, is important to give access to an adequate treatment planning and to guarantee at least the administration of palliative chemotherapy, with the aim for disease control and possible increased survival.

## Data Availability

Data and materials related to this case report are retained by the corresponding author.

## Abbreviations

AS: Angiosarcoma
AAA: Abdominal Aortic Aneurysm
CT: Computed tomography
EA: Epithelioid angiosarcoma
MRI: Magnetic resonance imaging
PTFE: Polytetrafluoroethylene
RBCs: Red blood cells
US: Ultrasound
